# A Non-Toxic Local Anæsthetic

**Published:** 1897-02

**Authors:** W. H. Jones

**Affiliations:** Fultonville, N. Y.


### A NON-TOXIC LOCAL ANÆSTHETIC.

BY W. H. JONES, D.D.S., FULTONVILLE, N. Y.

Even the most conservative practitioner finds a non-toxic and effective local anæsthetic a grateful addition to the dental armamentarium. The greater number of our patients have often heard of having a tooth extracted painlessly, and as there are always occasions when a tooth has reached a condition where it is better out of the mouth than in it, so, when extraction becomes a necessity, the operator will be asked the question, Can you not put something on the gums to alleviate the pain?

The hypodermic injection of eucaine, combined as follows, will give a satisfactory and painless extraction, and the nervous and excited patient will never again postpone a necessary extraction, but will endure the next operation, should one be necessary, with unruffled nerves:

| | Grammes. |
|---|---|
| Eucaine hydrochlorate | 0.8 |
| Hamamelis Virginiana | 7.8 |
| Glycerole | 1.6 |
| Hydronaphthol sol. | 0.129 |
| Guaiacol | 0.129 |
| Strophanthin | 0.013 |
| Aquæ dest. | 19.5 |
| Saccharin ad grat. | |

The above formula has been used with unusual success for the past six months. There are many cases in which the operator does not care to administer nitrous oxide or ether, but owing to the nervous and sometimes debilitated condition does not wish to operate without alleviating the pain in some manner. A local anæsthetic is a useful friend in this extremity.

The point of puncture can be anæsthetized by touching the mucosa with a solution of trichloracetic acid, glycerol, and cocaine in the following proportions:

| | Grammes. |
|---|---|
| Trichloracetic acid | 1.296 |
| Glycerol | 1.944 |
| Cocaine | 1.620 |

If the gums are healthy, they are not very sensitive, and the puncture may be made by making a firm pressure with the index-finger of the left hand and placing the needle at the edge of the point of pressure. Disinfect the mucosa before proceeding to operate.

